# Candidate Gene Identification for Systemic Lupus Erythematosus Using Network Centrality Measures and Gene Ontology

**DOI:** 10.1371/journal.pone.0081766

**Published:** 2013-12-02

**Authors:** Bhaskara Rao Siddani, Lakshmi Priyanka Pochineni, Manimaran Palanisamy

**Affiliations:** 1 C R Rao Advanced Institute of Mathematics, Statistics and Computer Science, Hyderabad, India; 2 Department of Biotechnology and Bioinformatics, University of Hyderabad, Hyderabad, India; Huazhong University of Science and Technology, China

## Abstract

Systemic lupus erythematosus (SLE) commonly accredited as “the great imitator” is a highly complex disease involving multiple gene susceptibility with non-specific symptoms. Many experimental and computational approaches have been used to investigate the disease related candidate genes. But the limited knowledge of gene function and disease correlation and also lack of complete functional details about the majority of genes in susceptible locus, encumbrances the identification of SLE related candidate genes. In this paper, we have studied the human immunome network (undirected) using various graph theoretical centrality measures integrated with the gene ontology terms to predict the new candidate genes. As a result, we have identified 8 candidate genes, which may act as potential targets for SLE disease. We have also carried out the same analysis by replacing the human immunome network with human immunome signaling network (directed) and as an outcome we have obtained 5 candidate genes as potential targets for SLE disease. From the comparison study, we have found these two approaches are complementary in nature.

## Introduction

Systemic lupus erythematosus (SLE) is a polygenic and multi-factorial disease, which often manifests different clinical phenotypes [1]. The conventional therapies for SLE include anti-malarial drugs, immunosuppressive medications, non-steroidal anti-inflammatory drugs (NSAIDs), which are non-specific and immunosuppressive. So the research is concentrated on developing the targeted therapies. Investigation of genetic predisposition through gene expression profiling and linkage analysis in multiple populations generates large sets of potential candidate genes. This approach successfully predicts genes for the diseases that cause a single risk, but fails to identify the genes causing complex disease [2]. This necessitated the development of in silico approaches such as ontology based, computation-based, and text based for the analysis of complex diseases [3]. In silico methods utilize the information of protein interactions, GO terms, gene expression data, sequence features, protein domains, protein function, orthologous connections, chromosomal regions, pathways, mutations (SNPs), chemical components, disease probabilities etc for predicting the candidate gene.

Recently, several online tools have been developed for prioritizing candidate genes, which usually combine the different in silico approaches [4], [5]. For example, SUSPECTS [6] ranks genes by matching sequence features, GO terms, interpro domains, and gene expression data. ToppGene [6] uses functional annotations, protein interaction networks to prioritize disease specific genes. Different tools like Polysearch [7], MimMiner [8], and BITOLA [9] relies on biological data mining. Posmed, a computational based approach prioritizes candidate genes using an inferential process similar to artificial neural network comprising documentrons [10]. Some tools like Phenopred use disease phenotype information which associate data from gene-disease relations [11], protein-protein interaction data, protein functional annotation at a molecular level and protein sequence data to detect novel gene-disease associations in humans. All these online tools have been successfully used for the prediction of candidate gene in diseases like epilepsy [12], osteoporosis [13], type II diabetes [14] and gene prioritization, depending on information of chromosomal location or genes differentially expressed in a tissue. But the above approaches have failed in case of SLE as it involves genes of differential expression patterns in tissues, influenced by various environmental factors. The limited information about the markers of SLE also contributed to their failure [15].

In such a situation, the network centrality measures coupled with the ontological terms favoured the identification of candidate genes for SLE. In the recent past many network based analysis have been developed for protein function prediction, identification of functional modules, classification of essential genes, synthetic lethality and disease candidate gene prediction etc. [16]–[24]. With the advances of sophisticated technologies for the functional annotation of genes, the candidate genes prioritization has become increasingly facile. GO terms are used for the systematic annotation of genes.

In the present work, we study the human immunome networks obtained through protein interaction network (undirected) and human signaling network (directed), in combination with the graph theoretic centrality measures and GO terms in order to identify candidate genes for SLE disease. For this purpose we have adopted the procedure developed by Csaba Ortutay *et. al*. [25]. In our work, we have applied more number of centrality measures such as eccentricity, centroid values, SP-betweenness, CF-closeness, CF-betweenness, Katz Status, Eigen vector and PageRank apart from closeness and degree centrality used by Csaba Ortutay *et. al*. This study effectively identifies the disease related candidate genes because of the consideration of more graph centrality measures. This approach can be applied to investigate other immune diseases for which sufficient information is present in databases.

## Materials and Methods

### Collection of Immunome proteins and construction of Human immunome networks

The immunome proteins were collected from the Immunome database [26] which consists of 893 human proteins along with their gene annotations. Similarly, we have collected the Human protein-protein interaction data from Human Protein Reference Database (HPRD) [27] which consists of 9617 proteins and 39,240 interactions. By mapping the immunome genes with the Human protein interaction data, we have extracted a sub-network named Human immunome network (undirected network). After removal of all orphan nodes, the core human immunome network consists of 548 proteins with 1372 interactions and which was used for further analysis.

### Immunome related SLE disease genes

From the Auto-immune disease database [28], 1402 SLE related proteins were collected, which contains not only the Human SLE genes but also microbial proteins as this disease is influenced by microbes. The microbial proteins were removed by comparing with human proteins taken from HPRD; as a result 1057 human related SLE genes were obtained. By comparing the human related SLE genes with immunome genes collected from the Immunome database, we found that only 329 genes were common and it is assumed to be known SLE genes (undirected network).

### Network properties and centrality measures

The scale-free nature of the human immunome network and other topological properties such as average degree, degree exponent, diameter, average clustering coefficient were calculated. The importance of the nodes in a network can be quantified through the concept of centrality, which allows ranking of the nodes based on their network structure. Various centrality methods have been developed based on the concepts of connectivity, distance, current flow and feedback. In our study, we have used 10 graph centrality measures namely degree, eccentricity, closeness, centroid values, shortest-path betweenness, current-flow closeness, current-flow betweenness, Katz status index, Eigen vector, PageRank [29]. We have calculated the above centrality measures for Human Immunome Network using the tool CentiBIN [30].

#### Degree centrality

The degree centrality measure ranks the potential of an individual node in the network based on its connectivity. 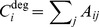
(1)


Here ‘A’ represents the adjacency matrix.

#### Eccentricity

Eccentricity centrality ranks the potential of an individual node ‘i’ based on the inverse of the maximum shortest path length that a particular node takes to reach rest of the nodes in the network. 
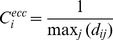
(2)


Here ‘d_ij_’ denotes the length of a shortest path between the nodes i and j.

#### Closeness centrality

**T**he potential of an individual node ‘i’ is calculated based on how closely it is located to other nodes in the network. Thus the inverse of the sum of the minimal path required to traverse from node ‘i’ to all other nodes in the network was used for ranking. 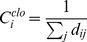
(3)


#### Centroid values

The centroid value of a individual node ‘i’ is calculated by considering the number of nodes that have minimum shortest path that are closer to ‘i’ than ‘j’. 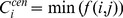
(4)


Where f (i, j): = γ_i_(j)−γ_j_(i) and γ_i_(j) denotes the number of node that are closer to i than j.

#### Shortest-path betweenness

The shortest path betweenness centrality ranks the potential of an individual node ‘i’ based on its ability to control signalling between any two nodes ‘j’ and ‘k’ in the network. 
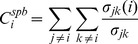
(5)


Here 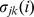 is the number of shortest paths between nodes ‘j’ and ‘k’ passing through the node ‘i’ and 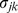 is the total number shortest paths between ‘j’ and ‘k’.

#### CF-Closeness

The current flow closeness centrality ranks the potential of an individual node ‘i’ based on the concept of Kirchhoff's law in an electrical network. The centrality score for a node ‘i’ is calculated from the distance between two vertices ‘i’ and ‘j’ which is defined as the difference of their electrical potentials. 
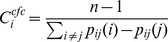
(6)


Where P_ij_ (i)–P_ij_(j) is the measure of distance between ‘i’ and ‘j’ in a network.

#### CF-Betweenness

The current-fiow betweenness centrality ranks the potential of an individual node ‘i’ based on the amount of information flow through node ‘i’ averaged over the nodes all pairs of ‘j’ and ‘k’. 
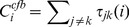
(7)


Here τ_jk_ (i) equals the fraction of information flow running over node ‘i’.

#### Katz status

Katz status centrality ranks the potential an individual node ‘i’ by considering its direct as well as indirect connections with the nodes in the network. Katz status centrality can be regarded as a generalization of the degree centrality. 
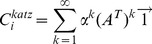
(8)


Where α is a positive constant, A^T^ is the transpose of adjacency matrix and ‘k’ is the degree of the node ‘i’.

*Eigen vector centrality:* Eigenvector centrality ranks the potential of the individual nodes in the network through the Eigen vector elements of the largest Eigen value λ of the network. 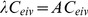
(9)


#### PageRank

PageRank centrality measure ranks the potential of an individual node based on the concepts of the algorithm used internet search engine. 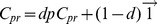
(10)


Where P is the transition matrix and d is the damping factor.

### Correlation analysis of centrality measures

The above centrality measures were calculated for each protein in the immunome network (undirected) and were ranked based on their scores. The correlation between different centrality measures were obtained using Spearman's rank correlation coefficient, *ρ* which is defined as 
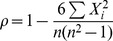
(11)


Here, differences 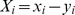 between the ranks of each observation on the two variables (centrality measures).

### GO terms enrichment and Predication of SLE candidate genes

The GO terms for all the 548 human immunome network genes were collected from Gene Ontology website ((http://www.geneontology.org) [31]. Using the Gene Ontology online tool, GOrilla [32] the gene enrichment was performed with the known SLE gene list as target and human immunome network genes as source using a p-value of 0.001. Significantly enriched GO terms were identified by using the threshold on the number of genes for each GO term i.e. the GO terms which were annotated for at least three but not for more than 50 immunome genes were considered. For each significantly enriched GO term of Biological Process, Molecular Function and Cellular Component, the genes matching the corresponding GO terms of the Human immunome network were identified as the list of genes with specific disease ontologies.

The lists of the genes with the 50 highest scores for each of the ten centrality measures were collected for the Human immunome network and the pooled list contains 113 genes. The significant SLE related GO terms were combined with the results in order to predict the new SLE related genes. A Venn diagram was used in order to represent high network score genes, genes with significant disease ontologies and known SLE genes. The genes that were present in both the lists of high network scores and significant disease ontologies but were not in the list of known SLE genes were predicted as novel SLE candidate genes.

## Results

### Topological properties of Human Immunome Network

We have constructed the human immunome network (undirected) from the data collected from the HPRD [27] and the immunome database [26]. The core immunome network contains 548 proteins with 1372 interactions. In this work, we have adopted the method developed by Csaba Ortutay to predict the SLE related candidate genes [25]. Csaba Ortutay and co-workers in their work used three centrality measures such as degree, closeness and efficiency for the prediction of primary immunodeficiency (PID) related genes. In our study for undirected network analysis, we have applied ten centrality measures to find the potential of individual proteins from the network [30]. From the **Table: 1** which shows the Spearman correlation coefficients for all pairs centrality measures, we conclude that the centrality measures of the undirected immunome network are not coincidental. The topological properties like the degree distribution that follows power law behavior (**γ** = 1.86), the average degree is 4.995 and the diameter is 12 were also calculated.

**Table 1:** The correlation coefficients of different pairs of centrality measures shows that the ranking of the nodes differs based on their formalism.

Centrality measures	Degree	Eccentricity	Closeness	Centroid Value	SP-betweenness	CF-closeness	CF-betweenness	Katz Status	Eigen vector	PageRank
**Degree**	1	0.56	0.69	0.88	0.85	0.94	0.93	0.96	0.68	0.93
**Eccentricity**	0.56	1	0.84	0.69	0.55	0.69	0.60	0.66	0.73	0.45
**Closeness**	0.69	0.84	1	0.81	0.65	0.82	0.69	0.82	0.94	0.55
**Centroid Value**	0.88	0.69	0.81	1	0.78	0.92	0.88	0.90	0.73	0.79
**SP-betweenness**	0.85	0.55	0.65	0.78	1	0.75	0.93	0.81	0.57	0.88
**CF-closeness**	0.94	0.69	0.82	0.92	0.75	1	0.86	0.97	0.81	0.81
**CF-betweenness**	0.93	0.60	0.69	0.88	0.93	0.86	1	0.88	0.61	0.93
**Katz Status**	0.96	0.66	0.82	0.90	0.81	0.97	0.88	1	0.82	0.87
**Eigen vector**	0.68	0.73	0.94	0.73	0.57	0.81	0.61	0.82	1	0.52
**PageRank**	0.93	0.45	0.55	0.79	0.88	0.81	0.93	0.87	0.52	1

### Gene enrichment analysis of top 50 high score genes

In order to verify the relevance of chosen centrality measures in identifying important immunome related GO terms, the gene enrichment analysis has been carried out for top 50 high score genes of each centrality measures. Enzyme linked receptor protein signaling pathway (GO: 0007167) and regulation of cytokine-mediated signaling pathway (GO:0001959) were dominant in majority of the centrality measures for biological process which indicates that signaling related ontology terms dominated the Biological process. The molecular function GO terms were dominated by the kinase activity among which Protein kinase activity (GO: 0004672), protein tyrosine kinase activity (GO: 0004713) and non-membrane spanning protein tyrosine kinase activity (GO: 0004715) were more significant. The cellular components were dominated by the membrane proteins (GO:0045121). From the above results, we can say that the centrality measures help in identification of important immunome related GO terms.

### Gene enrichment analysis of known SLE genes

The gene enrichment analysis was carried out for the known SLE genes with p-value <0.001. In case of molecular function only growth factor activity (GO: 0008083) was observed and for cellular components no GO terms were enriched. The regulatory process such as positive regulation of B-cell activation, proliferation (GO:0050871, GO:0030890), positive regulation and regulation of chemotaxis (GO:0050921, GO:0050920), regulation of immunoglobulin production (GO:0002637), regulation of T-cell differentiation and proliferation (GO:0045580, GO:0042129) etc., dominated the biological process gene ontology terms of known SLE genes. Most of the significantly enriched GO terms of known SLE are highly related to the immune process (**Table S1**).

### Prediction of new SLE candidate genes

**Figure 1:**
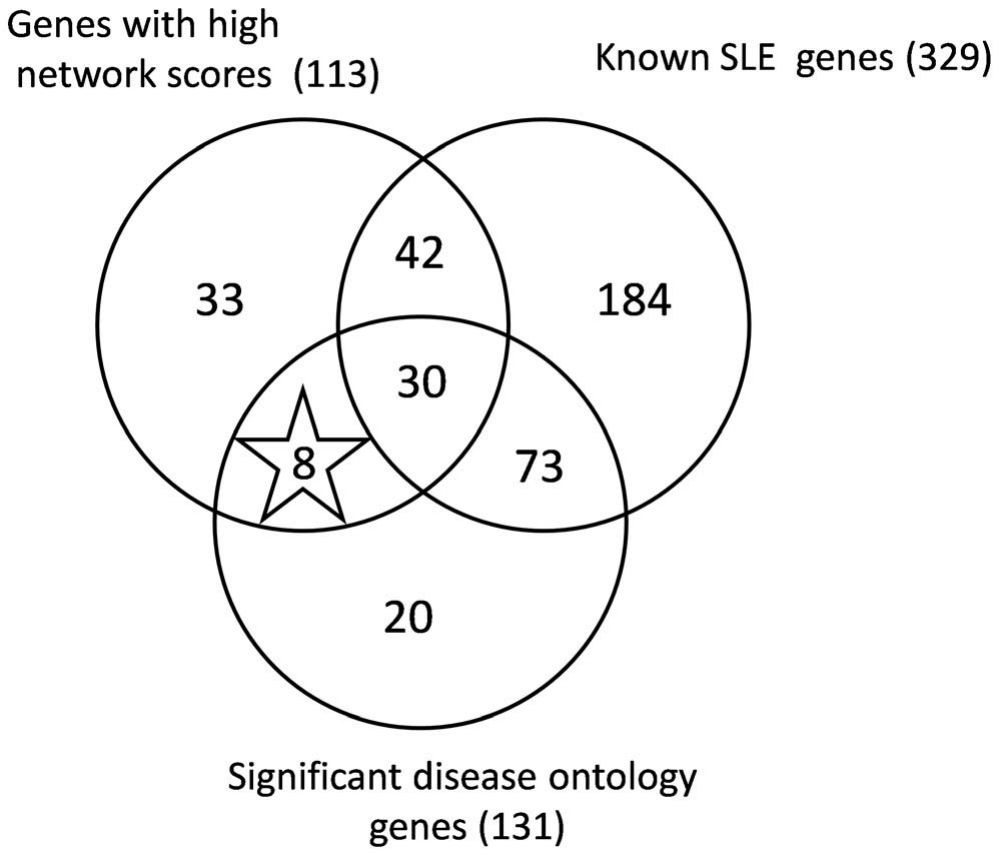
Venn diagram of candidate gene prediction, Genes with high network scores having significant GO terms are 38, in that gene set, 30 genes are already known as SLE related genes, so the remaining 8 genes are predicted as new SLE related candidate genes.

We have combined the gene lists for the high network scores, known SLE genes and genes with significant disease ontologies in order to predict the novel SLE genes. By combining, the lists of the genes with top 50 high scores for all the centrality measures, we obtained 113 unique genes out of which72 are known SLE genes. From the GO enrichment analysis we obtain 131 genes with significant disease ontologies out of which 103 were known SLE genes. Altogether 145 of the 329 the known SLE genes were selected either by GO terms or high scores of centrality measures. We found 38 genes had both high scores and significant GO terms in those 30 genes are known SLE genes and the same is clearly represented as a Venn diagram **Figure: 1** and the details of the predicted genes are given in the **Table: 2**.Thus we were able to identify 8 genes as novel SLE candidate genes such as HSP90B1, IGF1R, PDGFRA, PDGFRB, SOCS3, TIRAP and YWHAZ.

**Table 2:** List of 8 predicted novel SLE related candidate genes.

Symbol	Full name	Gene ID
**HSP90B1**	Heat shock protein 90 kDa beta	7184
**IGF1R**	Insulin-like growth factor 1 receptor	3480
**LCP2**	Lymphocyte cytosolic protein 2	3937
**PDGFRA**	Platelet-derived growth factor receptor, alpha polypeptide	5156
**PDGFRB**	Platelet-derived growth factor receptor, beta polypeptide	5159
**SOCS3**	Suppressor of cytokine signaling 3	9021
**TIRAP**	Toll-interleukin 1 receptor (TIR) domain containing adaptor	114609
**YWHAZ**	Tyrosine 3-monooxygenase/tryptophan 5-monooxygenase activation protein, zeta polypeptide	7354

The annotation for the predicted disease related candidate genes along with their relevance to immune system activities was obtained from EntrezGene database [33] and is given as follows; **HSP90B1:** Expression of this gene is associated with a variety of pathogenic states. HSP90B1 is heat shock protein 90 kDa beta (Grp 94), member 1. It is involved in various immune system activities such as innate immune response, negative regulation of apoptotic process etc. **IGF1R:** Insulin like growth factor 1 receptor, functions as an anti-apoptotic agent by enhancing cell survival. It plays a crucial role in immune system functioning viz responses, negative regulation of apoptotic process. **LCP2:** Lymphocyte cytosolic protein also known as SLP76 was found to play a positive role in promoting T-cell development and activation as well as mast cell and platelet function, and in innate immune and immune responses. **PDGFRA:** Platelet derived growth factor receptor, alpha polypeptide plays a role in wound healing, which is necessarily an immune system activity and also it plays an important part in innate immune responses. **PDGFRB:** Platelet derived growth factor receptor, beta polypeptide participate in cellular response to platelet derived growth factor stimulus, innate immune responses. **SOCS3:** Suppressor of cytokine signaling is negative regulator of cytokine signaling and participates in various immune processes such as protein ubiquitination, negative regulator of apoptotic process etc. **TIRAP:** Toll – interleukin 1 receptor domain containing adaptor protein is involved in the TLR4 signaling pathway of immune system. It actively participate inflammatory, immune responses, negative regulation of growth of symboint in host which account to immune system functions. **YWHAZ:** this Zeta polypeptide actively participates in apoptotic process, platelet activation, negative regulation of apoptotic process etc which accounts to immune system activities.

It is worth emphasizing that Csaba Ortutay and his coworkers have used only three centrality measures for predicting the candidate genes. But in our study, we have used ten centrality measures, keeping in mind that each centrality measures ranks the nodes based on their formalism which is different from each other. The Spearman rank correlation is evident that each centrality measure ranks the proteins in the network independently. The ten centrality measures and the corresponding ranks of all 8 novel genes were given in **Table-3**.

**Table 3:** Ranking of predicted candidate genes in various centrality measures in the order Degree-1, Eccentrity-2, Closeness-3, Centroid values-4, SP betweenness-5, CF closeness-6, CF betweenness-7, Katz status-8, Eigen vector-9 and PageRank-10.

Predicted candidate genes	Centrality measures (Ranking)
**HSP90B1**	2(28)
**IGF1R**	3(36), 4(29), 6(48), 8(45), 9(24)
**LCP2**	2(43), 3(43), 9(42)
**PDGFRA**	9(30)
**PDGFRB**	2(41), 3(19), 4(24), 6(27), 8(22), 9(14)
**SOCS3**	1(48), 4(43), 6(34), 8(34), 9(18)
**TIRAP**	4(50), 5(46)
**YWHAZ**	7(44), 10(50)

The rank of each gene is given inside the braces of the corresponding centrality measures in the top 50 rankings.

### Candidate gene prediction through human signaling network: A comparison study

We have carried out the candidate gene prediction analysis by replacing the human protein protein interaction network (undirected) with human signalling network (directed). For this purpose, we have used the human signalling network from the database developed by Edwin Wang [24]. The network contains of 6309 proteins and 62737 interactions that includes activation ‘+’, inhibition ‘−’ and physical interaction ‘0’. The human immunome signalling network (HISN) was obtained by mapping the immunome genes with human signalling network. The core HISN contains 304 proteins with 627 interactions. The directions were assigned based on the type of interaction, for the physical interaction the signaling can happen in two directions (i.e. the signaling between protein A to B and B to A), for activation and inhibition signaling through one direction (i.e. if protein A activates protein B, the signaling direction from A to B and if protein A inhibits protein B, the signaling direction is from B to A).

**Figure 2:**
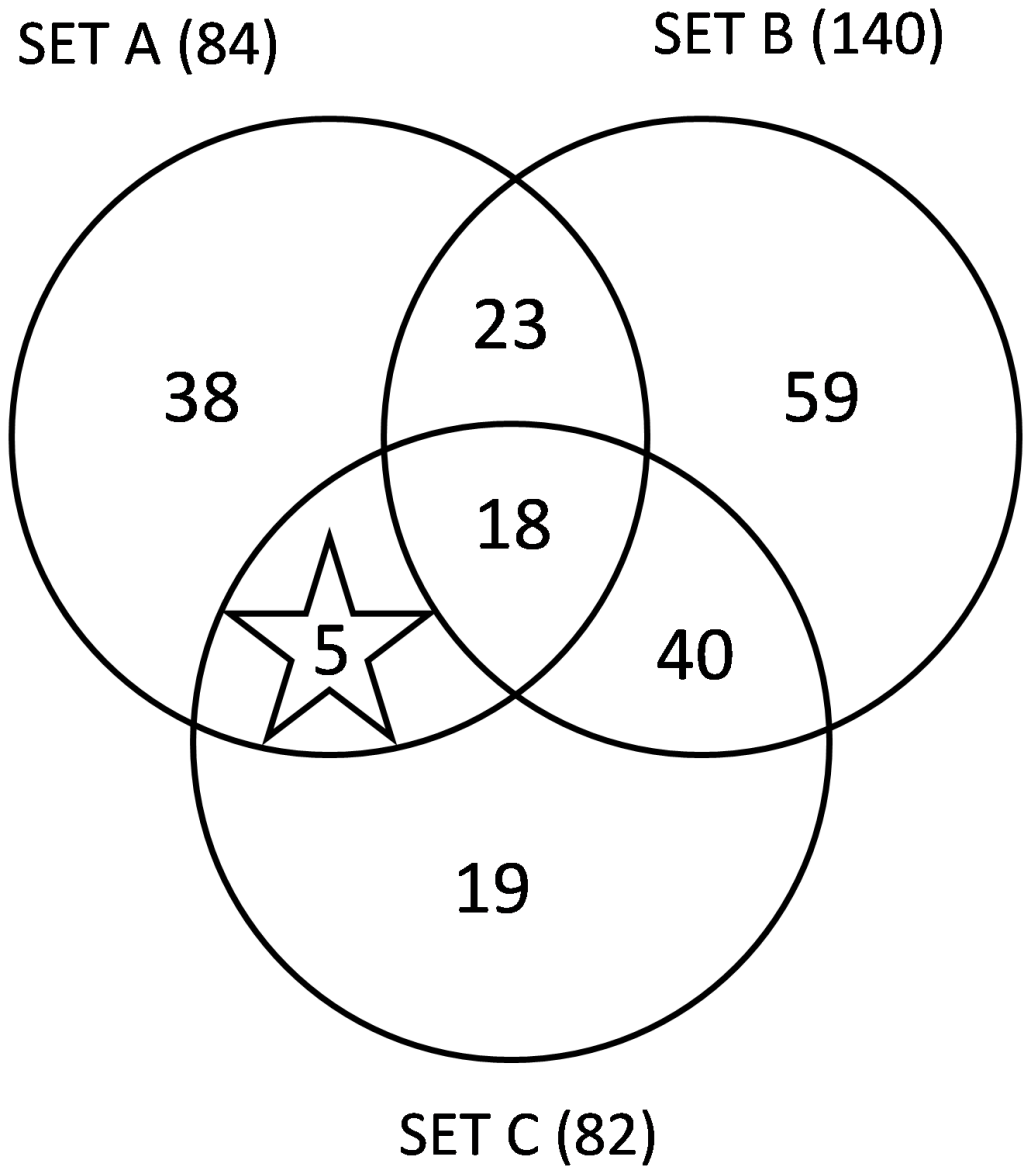
Venn diagram of candidate gene prediction using human signally network, SET A refers the genes with high network scores, SET B refers the Known SLE genes and SET C refers genes with significant disease ontologies. Gene with high network score having significant GO term are 23, in that 18 are already known as SLE related genes, so the remaining 5 genes are predicted as new SLE related candidate genes.

For the human immunome network, the ten centrality measures were calculated to rank the potential of the individual proteins in the network. Since the HISN is sparse the CentiBiN tool was able to calculate only the in-degree, out-degree, Katz status and PageRank measures. After pooling the top 50 ranking genes, we have obtained 87 genes (SET A) which are highly essential/hub genes of the HISN. The known 140 SLE (SET B) genes were extracted by mapping the HISN genes with 1024 SLE related genes collected from Auto-immunome disease database. The gene enrichment analysis were performed by keeping Known SLE genes as target and all HISN genes as source using the Gorilla online tool. As a result, we have found 84 genes with significant disease ontologies (SET C). Using the SET A, B & C genes list, we have predicted 5 genes such as NFKBIA, NCF2, TIRAP, IL18BP and XCR1 as potential targets for SLE disease (**Figure: 2**). Also we found only TIRAP gene is common among the candidate genes obtained through both human immunome network and human immunome signaling network analysis.

## Discussion

Human Protein-interaction networks have been used so far for the identification of genes responsible for causing disease and prediction of new disease candidate genes like PID, type II diabetes, osteoporosis, and neuro-degenerative diseases for drug discovery [34]–[37]. A recent review focuses on prediction of the protein networks and identification of the target genes that can be used for drug discovery [38]. Several Insilico methods are being used to reduce the cost of disease gene prioritization. For this, protein interaction networks were preferably used rather than other biological networks.

One can understand the functional importance of proteins from the network through different centrality measures. Centrality measures ranks network elements according to their importance within the network structure. In our study, we have used ten centrality measures to identify bottleneck proteins in the immunome network. The advantage of using 10 different centrality measures is that different centrality measures focuses on different important concepts and rank nodes differently based on their types such as connectivity, Distance, Shortest-Path, Current-Flow, and Feedback. The biological relevance of the proteins can be extracted through GO terms, which provide information for protein functions, processes and localization. Our approach combines the ten centrality measures and the GO terms to find the potential genes that are responsible for SLE disease. We have used CentiBiN tool to calculate the centrality measures for the Human Immunome protein protein interaction network. The tool was designed in such a way that it calculates 17 different centrality measures. The tool CentiBiN was able to calculate the scores for only 13 centrality measures because of the large size of our interaction network and hence the rest 4 measures were discarded. The pairwise correlation coefficients for all those13 centrality measures were calculated. We have found that the correlation coefficient among certain centrality measures such as ‘radiality’, ‘closeness’ and ‘HITS’, ‘Eigen vector’ is 1. Also we have observed that ranking of the nodes based on radiality is same as that of ‘closeness centrality’ and same is the case with that of HITS and Eigen vector centralities. Similarly, the correlation coefficient of “Bargaining centrality” with other centrality measures is in negative, which indicates that it is highly anti-correlated with other centrality measures. So, based on the implications of the aforementioned analysis, we have decided to exclude ‘HITS’, ‘Radiality’ and ‘Bargaining’ centrality measure in our work.

**Figure 3:**
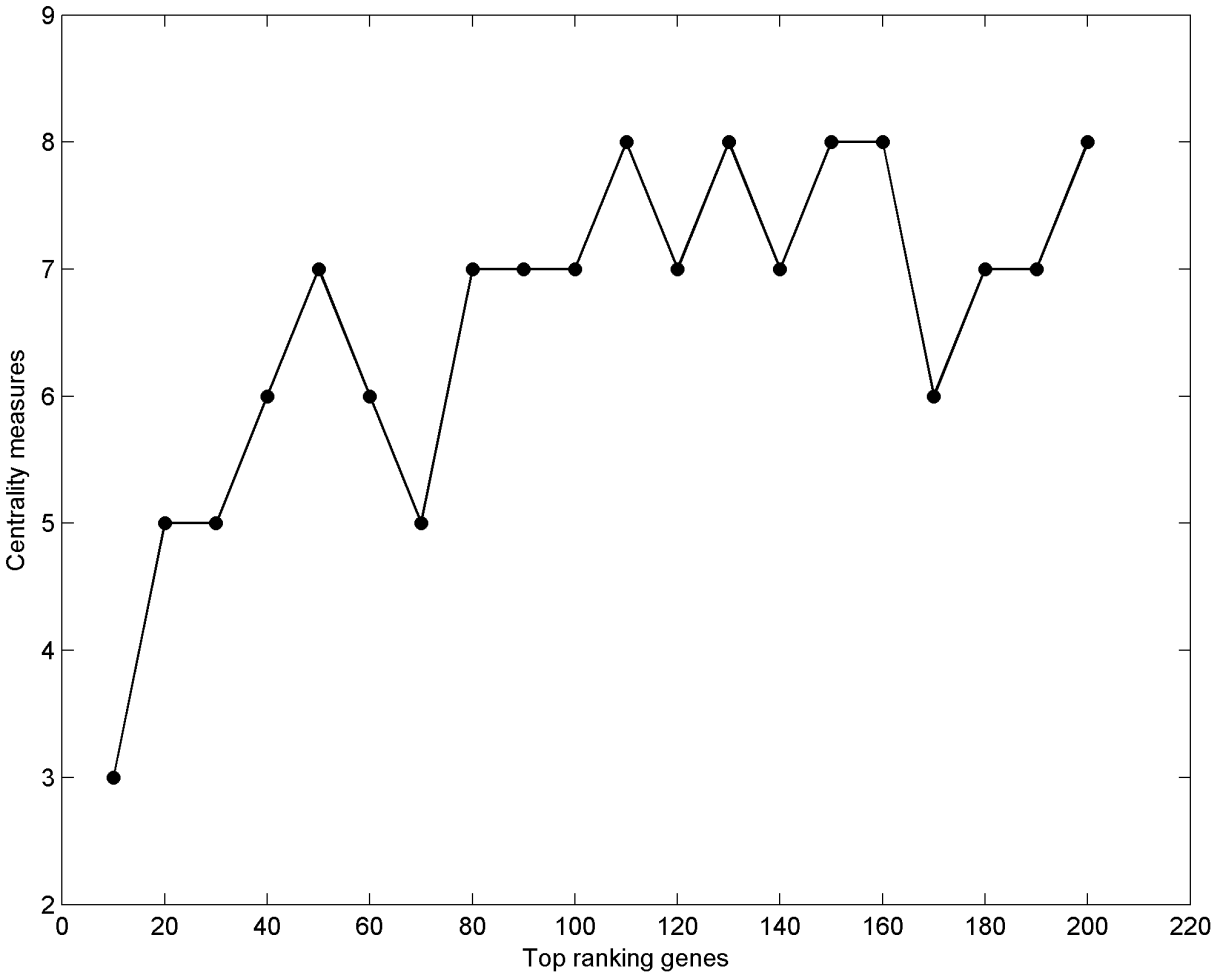
Depending on the number of genes that are considered as top ranking genes, the numbers and combinations of centrality measures may vary for network analysis.

In general, centrality measures ranks the potential of individual nodes in the network and the ranking of nodes considerably differ for each of centrality measure. In our work the position of the node in terms of its rank is negotiable as we have considered and pooled out top 50 ranking genes. The genes that persist of top 50 scoring genes for degree, CF-closeness, Katz status and PageRank centrality are considerably same. Hence any one of the above 4 centrality measures can be considered along with other six centrality measures like eccentricity, closeness, centroid values, SP-betweenness, CF-betweenness and Eigen vector. Thus in total seven centrality measures are sufficient for our study and it also worth emphasizing that combinations of various centrality measures differs with the number of genes that are considered as top ranking, this is clearly shown in the **Figure: 3**.

In the present study from the undirected human immunome network analysis, we have predicted 8 novel SLE-related candidate genes, which are found to have numerous functions and several interaction partners of the immune system. These genes were further considered for validation and from the literature survey we have observed that among the 8 predicted genes, 3 genes SOCS3, TIRAP and PDGFRA were found to act as specific markers for the SLE disease [39]–[42].

Similarly from directed human immunome signaling network analysis, we have predicted 5 novel genes which have direct relevance as candidate genes for SLE. The five genes predicted NFKBIA, NCF2, TIRAP, IL18BP, XCR1 were analyzed through literature for their relevance as candidate genes for SLE. The gene nuclear factor kappa B, NFKBIA is an important transcription factor participates in the activation of genes involved in immune responses. The work of Romzova and his coworkers proved the association of NFKBIA with SLE through genotyping [43]. Jacob and his coworkers reported a multidisciplinary approach resulting in the identification of neutrophil cytosolic factor 2 (NCF2) as an important risk factor for SLE and proved that NCF2 is strongly associated with increased risk of SLE [44]. One of the intent elementness of the study conducted by Castiblanco was to investigate the influence of the functional TIRAP (MAL) S180L polymorphism on the autoimmune disease systemic lupus erythematosus (SLE) and proved that it is a specific factor for SLE [45]. Overproduction of inflammation-related cytokines plays an important role in systemic lupus erythematosus (SLE), increased levels of both IL-18 and its natural inhibitor IL-18BP, characterize SLE [46]and this proves the gene to be a candidate for SLE. The expression of the gene lymphocyte chemokine receptor (XCR1) in peripheral blood mononuclear cells (PBMC) of SLE and its correlation with disease was proven by Chinese scientists which elucidates its relevance as candidate gene for the disease [47].

Also, we have tried to identify the available drugs for the predicted SLE-related candidate genes using the online database DrugMapCentral, which is an unique informatics resource for translational studies on drugs (http://r2d2drug.org/DMC.aspx). But we found that no drugs have been discovered for these novel SLE-related candidate genes. The predicted novel SLE-related candidate genes can act as potential targets for the drug design and discovery because of their significant disease ontologies and high enrichment scores. The surface properties of target proteins can be studied by acquiring the structural information from PDB and one can identify the active site of the targets and design drugs for these targets.

From the undirected human immunome network analysis, we have predicted 8 SLE related candidate genes in which 3 genes has specific relevance to SLE disease based on reported research [39]–[42]. Also, we have observed that TIRAP gene is common with the 5 genes obtained through human immunome signaling network analysis, in which all genes have specific markers to SLE disease [43]–[47]. Therefore, these two methods are complementary and have their own merit in predicting new disease candidate genes. The approach we have employed in our study uses the information about the interactions among the proteins of the human immunome and also their functional annotation. This method may be further applied to other immune related diseases which are completely dependent on malfunctioning of immune system such as autoimmune pancreatitis, Graves' disease, multiple sclerosis, scleroderma.

## Supporting Information

Table S1 **Significantly enriched biological process GO terms of Known SLE genes.**
(DOC)
