# Supplementary material for: Candidate Gene Identification for Systemic Lupus Erythematosus Using Network Centrality Measures and Gene Ontology
Source: PLoS One. 2013 Dec 2;8(12):e81766. doi: 10.1371/journal.pone.0081766 (PMC3847089; doi:10.1371/journal.pone.0081766)
Supplement: Table S1 — Significantly enriched biological process GO terms of Known SLE genes. (DOC) [file pone.0081766.s001.doc]

**Table S1:** Significantly enriched biological process GO terms of Known SLE genes

| **S.no.** | **GO Term** | **Description** |
| --- | --- | --- |
|  | GO:0030595 | leukocyte chemotaxis |
|  | GO:0030168 | platelet activation |
|  | GO:0002576 | platelet degranulation |
|  | GO:0050871 | positive regulation of B cell activation |
|  | GO:0030890 | positive regulation of B cell proliferation |
|  | GO:0048520 | positive regulation of behavior |
|  | GO:0030335 | positive regulation of cell migration |
|  | GO:2000147 | positive regulation of cell motility |
|  | GO:0051272 | positive regulation of cellular component movement |
|  | GO:0050921 | positive regulation of chemotaxis |
|  | GO:0050729 | positive regulation of inflammatory response |
|  | GO:0002690 | positive regulation of leukocyte chemotaxis |
|  | GO:0002687 | positive regulation of leukocyte migration |
|  | GO:0040017 | positive regulation of locomotion |
|  | GO:0050671 | positive regulation of lymphocyte proliferation |
|  | GO:0050731 | positive regulation of peptidyl-tyrosine phosphorylation |
|  | GO:0050714 | positive regulation of protein secretion |
|  | GO:0042102 | positive regulation of T cell proliferation |
|  | GO:0022603 | regulation of anatomical structure morphogenesis |
|  | GO:0045765 | regulation of angiogenesis |
|  | GO:0050864 | regulation of B cell activation |
|  | GO:0030888 | regulation of B cell proliferation |
|  | GO:0050795 | regulation of behavior |
|  | GO:0030155 | regulation of cell adhesion |
|  | GO:0050920 | regulation of chemotaxis |
|  | GO:0070201 | regulation of establishment of protein localization |
|  | GO:0032844 | regulation of homeostatic process |
|  | GO:0002637 | regulation of immunoglobulin production |
|  | GO:0002688 | regulation of leukocyte chemotaxis |
|  | GO:0002685 | regulation of leukocyte migration |
|  | GO:0032880 | regulation of protein localization |
|  | GO:0050708 | regulation of protein secretion |
|  | GO:0051223 | regulation of protein transport |
|  | GO:2000241 | regulation of reproductive process |
|  | GO:0044057 | regulation of system process |
|  | GO:0045580 | regulation of T cell differentiation |
|  | GO:0042129 | regulation of T cell proliferation |
|  | GO:0034103 | regulation of tissue remodeling |
|  | GO:0009991 | response to extracellular stimulus |
|  | GO:0001666 | response to hypoxia |
|  | GO:0007584 | response to nutrient |
|  | GO:0031667 | response to nutrient levels |
|  | GO:0070482 | response to oxygen levels |
